# The Met Needs for Pediatric Surgical Conditions in Sierra Leone: Estimating the Gap

**DOI:** 10.1007/s00268-017-4244-8

**Published:** 2017-09-20

**Authors:** Carmen Mesas Burgos, Håkon Angell Bolkan, Donald Bash-Taqi, Lars Hagander, Johan Von Screeb

**Affiliations:** 1 Women’s and Children’s Health, Karolinska Institute, Stockholm, Sweden; 2 Department of Cancer Research and Molecular Medicine, Norwegian University of Science and Technology, Trondheim, Norway; 3 Ministry of Health and Sanitation, Freetown, Sierra Leone; 4 Surgery and Public Health, Pediatric Surgery, Department of Clinical Sciences in Lund, Skåne University Hospital, Lund University, Lund, Sweden; 5 Global Health-Health System and Policy Department of Public Health Sciences, Centre for Research on Health Care in Disasters, Stockholm, Sweden

## Abstract

**Background:**

In low- and middle-income countries, there is a gap between the need for surgery and its equitable provision, and a lack of proxy indicators to estimate this gap. Sierra Leone is a West African country with close to three million children. It is unknown to what extent the surgical needs of these children are met.

**Aim:**

To describe a nationwide provision of pediatric surgical procedures and to assess pediatric hernia repair as a proxy indicator for the shortage of surgical care in the pediatric population in Sierra Leone.

**Methods:**

We analyzed results from a nationwide facility survey in Sierra Leone that collected data on surgical procedures from operation and anesthesia logbooks in all facilities performing surgery. We included data on all patients under the age of 16 years undergoing surgery. Primary outcomes were rate and volume of surgical procedures. We calculated the expected number of inguinal hernia in children and estimated the unmet need for hernia repair.

**Results:**

In 2012, a total of 2381 pediatric surgical procedures were performed in Sierra Leone. The rate of pediatric surgical procedures was 84 per 100,000 children 0–15 years of age. The most common pediatric surgical procedure was hernia repair (18%), corresponding to a rate of 16 per 100,000 children 0–15 years of age. The estimated unmet need for inguinal hernia repair was 88%.

**Conclusions:**

The rate of pediatric surgery in Sierra Leone was very low, and inguinal hernia was the single most common procedure noted among children in Sierra Leone.

## Introduction

### The global burden of surgical conditions

Five billion people do not have access to safe and affordable surgical care when needed [1, 2]. The gap between the need for surgery and its equitable and safe provision is considerable. Although 75% of the world’s population lives in low- and middle-income countries (LMICs) [3], only 6% of all surgical procedures worldwide are estimated to be performed on the poorest 37% of the world’s population [2]. The Lancet Commission for Global Surgery emphasized the importance of surgery to achieve sustainable development and quantified a global minimum rate of 5000 annual surgical procedures per 100,000 population [4]. Currently, an additional 143 million surgical procedures are needed each year [2], and surgery could avert 1.5 million annual deaths [2], 6–7% of all avoidable deaths in low- and middle-income countries [5].

### Global pediatric surgery

Within the newly emerging field of global surgery, global pediatric surgery has recently gained more attention [6–8]. Studies indicate that congenital malformations account for 6% of all infant deaths, with 96% of the fatalities occurring in low- and middle-income countries [9], but overall, there is a lack of studies quantifying the burden surgical diseases and needs of surgical care in the pediatric population. Even if the majority of the deaths under 5 years of age are due to infectious diseases [9, 10], increased access to safe surgical care could save the lives of a significant number of children worldwide [11]. While the needs for pediatric surgery in low- and middle-income countries have not been documented in detail, studies have indicated that they are largely unmet [12–18]. The reasons for this are complex: a combination of factors such as variations in coverage, financial capacity, lack of trained staff and awareness in the population, in addition to limited political commitment [6, 7, 16, 17, 19–22]. Studies on the volumes of pediatric surgical procedures performed at individual facilities in low- and middle-income countries have highlighted the gap between the need for surgery and the equitable provision of safe surgical care in these settings [19, 20, 23–26]. In the adult population, proxy indicators such as hernia repair or cesarean section have been used to estimate equitable provision of surgical care [27], but similar proxy indicators in the pediatric population are lacking.

### Sierra Leone

The Republic of Sierra Leone is a West African low-income country with an estimated population of 5.9 million in 2012 [28], 42% being under the age of 15 years [29]. In 2012, the under-five mortality was among the highest in the world at 142 per 1000 live births [30]. There are well-documented shortages in infrastructure and supplies required to deliver safe surgical care in the country, with significantly less resources at governmental hospitals compared to private and mission hospitals [31]. The shortages in workforce have previously been documented: Including gynecologists, orthopedic surgeons, ophthalmologists and ENT specialists, there are only 58 (0.9/100,000 inhabitants) specialist surgical providers in the country [32, 33], which is 50 times less than recommended [2]. When trying to estimate the needs of surgical care, a household survey on self-reported surgical conditions concluded that lack of access to safe and affordable surgical care contributes to a significant excess mortality [15, 34]. Studies based on single institutions [21] or governmental hospitals [18] have highlighted deficiencies in the pediatric surgical capacity in the country. However, there is no comprehensive, quantified data on the met and unmet need of surgery in the pediatric population in Sierra Leone.

We hypothesized that provision of safe pediatric surgical care is insufficient, that surgical needs among Sierra Leonean children are largely unmet, and that hernia repair can be used to roughly estimate equitable provision of surgical care in children.

The aim of this study was to define and describe the nationwide provision of pediatric surgery in Sierra Leone, how much is performed, where and by whom. Further, we aimed to assess pediatric hernia repair as a proxy indicator for the provision of surgery in the pediatric population in Sierra Leone and to estimate the unmet need for hernia repair.

## Materials and methods

### Study design and setting

This nationwide retrospective facility-based survey of all surgeries performed in Sierra Leone in 2012 was a joint collaboration between the Sierra Leonean Ministry of Health and Sanitation (MOHS), the Non-Governmental Organization (NGO) CapaCare, the Norwegian University of Science and Technology (NTNU) and Karolinska Institute.

### Inclusion criteria

All facilities performing one or more of the 21 surgical procedures listed by the World Health Organization were included (“Appendix”) [35].

### Data collection

Data were collected between January and May 2013, from all facilities in the country where surgical procedures were performed. Data were collected from operating theater, anesthesia and delivery logbooks using methods that have previously been described [36]. From this data set, we obtained data on all surgical procedures for the pediatric population, defined as under the age of 16 years. We extracted data on: surgical provider and type of surgical procedures, geographical location, type and owner of institutions performing surgery, as well as patient characteristics.

### Primary and secondary outcomes

Primary outcomes were rate and volume of surgical procedures performed in children in 2012 in Sierra Leone, and independent variables were age, type of facility, workforce and district. Results were compared to previously reported results for the adult population [36]. Further, we calculated the expected number of inguinal hernia in children as a proxy indicator for the need of surgical care and estimated the unmet need of surgery among children between 0 and 15 years of age.

### Data analysis

#### Rate and volume of pediatric surgical procedures in Sierra Leone

Surgical rate was defined as number of surgical procedures per 100,000 population 0–15 years. The 2012 projections from the most recent census were used to calculate age-specific rates of surgery [28]. Surgical volume was defined as the number of surgical procedures performed annually and was reported as frequencies.

### Type of surgical procedure and age distribution

For all surgical procedures, gender and age (0–28 days, 29 days–11 months, 1–4 years, 5–15 years) were recorded, together with characteristics of the surgical procedures, such as type, urgency and date. To uniformly record the surgeries, 34 pre-defined categories of the most commonly performed procedures were created within each specialty (general surgery, orthopedic surgery, ophthalmic surgery, obstetrics and gynecology) (Tables 1, 2).

**Table 1:** Rate of the most commonly performed pediatric surgical procedures in Sierra Leone in 2012 divided by age-groups, per 100,000 inhabitants (population 0–4 years: 995,665, 5–15 years: 1,812,655)

	Procedures/100,000 0–4 years	Procedures/100,000 5–15 years	Procedures/100,000 0–15 years
*General surgery*	74	46	56
Hernia repair	25	10	16
Appendectomy	0.3	8	6
Laparotomy	5	8	7
Incision and drainage abscess	6	4	4
General cancer surgery	0.3	1	0.6
Urethral stricture dilatation	0.1	0.4	0.3
Chest tube	0.5	0.2	0.3
Neonatal surgery	2		0.6
Cystostomy	0.1	0.1	0.1
Tracheostomy	0.1	0.1	0.1
Cleft lip repair	0.1	0.1	0.1
General surgery other	34	14	21
*Obstetric and gynecology*	0	6	4
Cesarean section	0	4	3
Dilatation and curettage	0	0.4	0.3
Hysterectomy	0	0.1	0.1
Obstetric fistula repair	0	0.6	0.4
Salpingectomy ectopic pregnancy	0	0.1	0.1
Manual placenta removal	0	0.2	0.1
Cervical or vaginal laceration	0	0.1	0.1
Ob–Gyn other	0.1	0.4	0.8
*Orthopedic surgery*	16	16	17
Operative fracture treatment	3	2	3
Amputation lower limb	0.5	0.5	0.5
Conservative fracture treatment	1	1	1
Amputation upper limb	0.4	0.7	0.6
Orthopedic cancer surgery	0.3	0.1	0.1
Orthopedic surgery other	11	12	12
*Ophthalmic surgery*	1	3	3
Cataract surgery	1	3	3
*Neurosurgery*	0.7	0.1	0.3
*Missing*	2	4	3
Total	94	75	84

**Table 2:** Surgical volumes of the twenty-eight pre-defined groups of the most commonly performed pediatric surgical procedures in Sierra Leone in 2012, by age-groups

	1–28 days		29 days–11 months		1–4 years		5–15 years		Total 0–15 years	
	*n*	%	*n*	%	*n*	%	*n*	%	*n*	%
*General surgery*	32	97	139	81	547	76	829	57	1547	65
Hernia repair	2	6	48	28	195	27	189	13	434	18
Appendectomy	0	0	0	0	3	0.6	151	10	154	7
Laparotomy	6	18	12	9	31	4	136	9	185	8
Incision and drainage abscess	1	3	18	11	36	5	68	5	123	5
General cancer surgery	0	0	0	0	3	0.4	15	1	18	1
Urethral stricture dilatation	0	0	0	0	1	0.1	7	0.5	8	0.3
Chest tube	0	0	3	2	2	0.2	4	0.3	9	0.4
Neonatal surgery	16	49	2	1	0	0	0	0	18	1
Cystostomy	0	0	0	0	1	0.1	2	0.1	3	0.1
Tracheostomy	0	0	0	0	1	0.1	1	0.1	2	0.1
Cleft lip repair	0	0	1	0,6	0	0	1	0.1	2	0.1
General surgery other	7	21	55	32	274	38	255	18	591	25
*Obstetric and gynecology*	0	0	0	0	1	0.1	108	7	109	5
Cesarean section	0	0	0	0	0	0	75	5	75	3
Dilatation and curettage	0	0	0	0	0	0	8	0.6	8	0.3
Hysterectomy	0	0	0	0	0	0	1	0.1	1	0.1
Obstetric fistula repair	0	0	0	0	0	0	10	0.7	10	0.4
Salpingectomy ectopic pregnancy	0	0	0	0	0	0	1	0.1	1	0.1
Manual placenta removal	0	0	0	0	0	0	3	0.2	3	0.1
Cervical or vaginal laceration	0	0	0	0	0	0	2	0.1	2	0.1
Ob–Gyn other	0	0	0	0	1	0.1	8	0.6	9	0.4
*Orthopedic surgery*	1	3	18	11	145	20	393	20	557	23
Operative fracture treatment	0	0	0	0	30	4	143	10	173	7
Amputation lower limb	0	0	1	0.6	4	0.6	9	0.6	14	0.6
Conservative fracture treatment	1	3	1	0.6	8	1	13	0.9	23	1
Amputation upper limb	0	0	0	0	4	0.6	13	0.9	17	0.7
Orthopedic cancer surgery	0	0	0	0	3	0.4	1	0.1	4	0.2
Orthopedic surgery other	0	0	16	9	96	13	214	15	326	14
*Ophthalmic surgery*	0	0	4	2	8	1	59	4	71	3
Cataract surgery	0	0	4	2	8	1	59	4	71	3
*Neurosurgery*	0	0	7	4	0	0	1	0.1	8	0.3
*Missing*	0	0	4	2	20	3	65	5	88	4
Total	33	1.4	172	7.2	721	30.2	1455	61	2381	100

### Type of facility, workforce and geographical variations

Providers were categorized as governmental if owned by government, or private for-profit or nonprofit facilities. Facilities were defined as hospitals if they offered 24-h emergency inpatient care; others were defined as clinics. Hospitals were further divided into referral hospitals, when providing highly differentiated clinical services, or district hospitals. The workforce performing the surgical procedure was categorized according to the specialization level (specialist trained surgeon or obstetrician, medical doctor, nurse, or associate clinician) and nationality (Sierra Leonean, non-Sierra Leonean workforce). Data were aggregated by administrative districts for analyses.

### Pediatric inguinal hernia as a proxy indicator for the need of surgery and estimation of the unmet needs

Hernia repair in adults has been used as a proxy indicator to estimate the unmet surgical needs in the setting of LMIC [27]. In the absence of a validated indicator for the pediatric population, we used pediatric inguinal hernia as a tracer for the burden of pediatric surgical conditions. Pediatric inguinal hernia is a common surgical condition in children, with stable incidence at 10–20/1000 live births [37, 38], and a prevalence of 3–5% in term infants [39]. Ninety percent of the cases are boys; one-third will appear during the first 6 months of life [39, 40]. The consensus in the pediatric surgical community is to recommend a surgical correction at the time of diagnosis [37], to avoid complications, since the risk of incarcerated inguinal hernias is estimated to be 16%, with potentially devastating consequences.

Surgical need was defined as the estimated hernia disease burden. Based on a hernia incidence rate of 10–20/1000 live births and the number of children born in 2012 [41], we calculated the annual incidence of inguinal hernia in Sierra Leone in 2012 and the prevalence based on the population from 0 to 15 years of age. Met need for hernia repair was defined as the number of surgical procedures for pediatric hernia repair performed [42]. Unmet need for hernia repair was defined as the expected cases of hernia in the population less than 16 years that have not been corrected with surgery [42]. The unmet need was calculated by subtracting the actual number of surgical procedures for hernia repair performed in the pediatric population from the expected cases of inguinal hernia (incidence rate multiplied by number of children born in 2012 [41]), divided by the expected number of cases.

### Statistical analysis

Frequencies were reported for categorical variables. Continuous variables were reported as mean, median, range and rates (surgical procedures per 100,000 population). SPSS^®^ version 23 and PRISM 6 (Graphpad Software Inc.) were used for descriptive statistics, analyses and figures. Chi-square test was used to explore differences between groups, and significant level was set at *p* < 0.05.

### Ethical considerations

Two ethical committees, Sierra Leone Ethics and Scientific Review Committee and the Regional Committees for Medical and Health Research Ethics in Central Norway (2012/2187) granted ethical clearance for this study prior to the data collection.

## Results

**Fig. 1:**
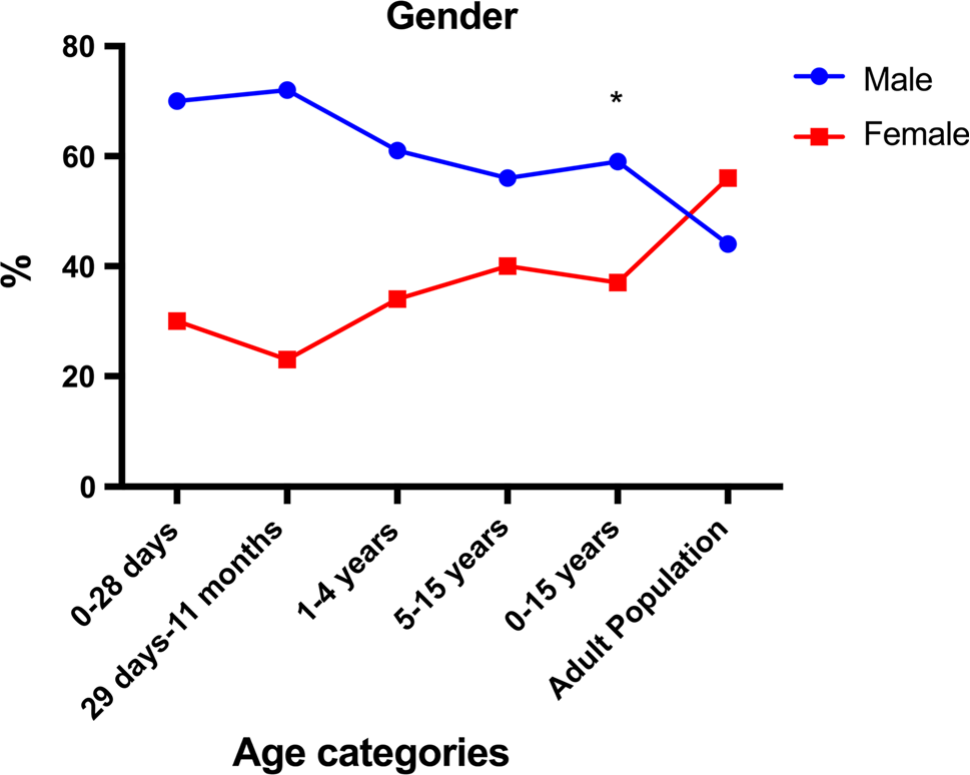
Gender distribution of surgical procedures in Sierra Leone in 2012 by age categories

A total of 2381 surgical procedures were recorded in children 0–15 years in Sierra Leone during 2012, corresponding to not more than 10% of all surgical procedures performed in the country that year. The rate of pediatric surgical procedures in Sierra Leone was 84 per 100,000 inhabitants 0–15 years of age (Table 1). The rate of surgery was higher in boys than in girls in all age-groups (Fig. 1) (*p* < 0.001). The rates of hernia repair ranged between 10 and 25/100,000, with higher rates at younger ages.

**Fig. 2:**
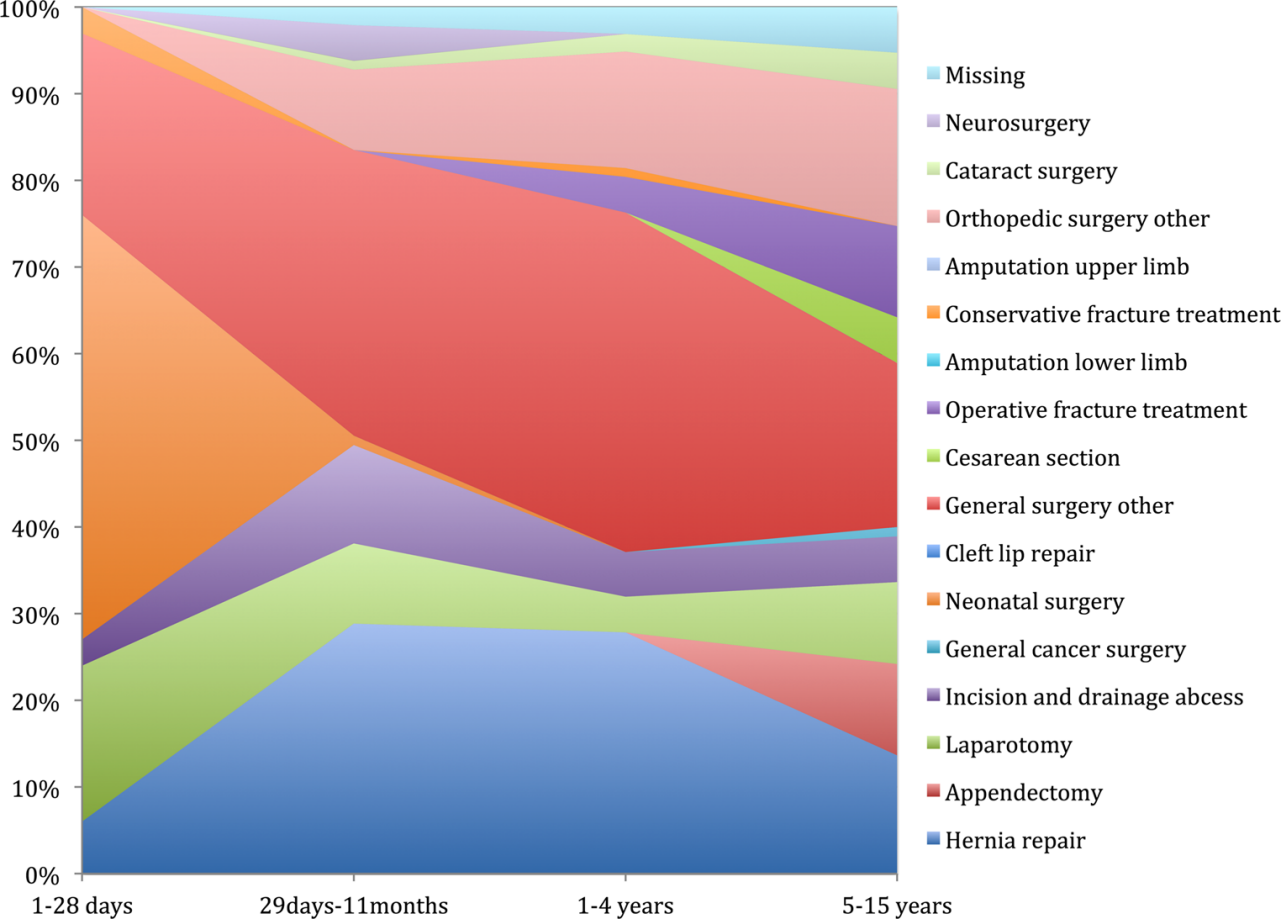
Surgical volumes of most common pediatric surgical procedures performed in Sierra Leone in 2012, by age categories

Forty-one percent (976) were registered as emergency surgeries, 15% (357) were registered as elective surgeries, and the remaining 44% (1048) were unknown. General surgical procedures represented 65% (1547) of the annual provision of surgical care, followed by orthopedic surgery (23%/557), obstetrics and gynecology (5%/109), and ophthalmic surgery (3%/71). Information was not available in 4% (88) of the cases (Table 2). The most common surgical procedure performed in children was hernia repair (18%/434), followed by laparotomy, appendectomy and open fracture management. Almost 40% of the surgical procedures were not specified; 25% of (591) those were general surgery procedures (Table 2, Fig. 2).

**Fig. 3:**
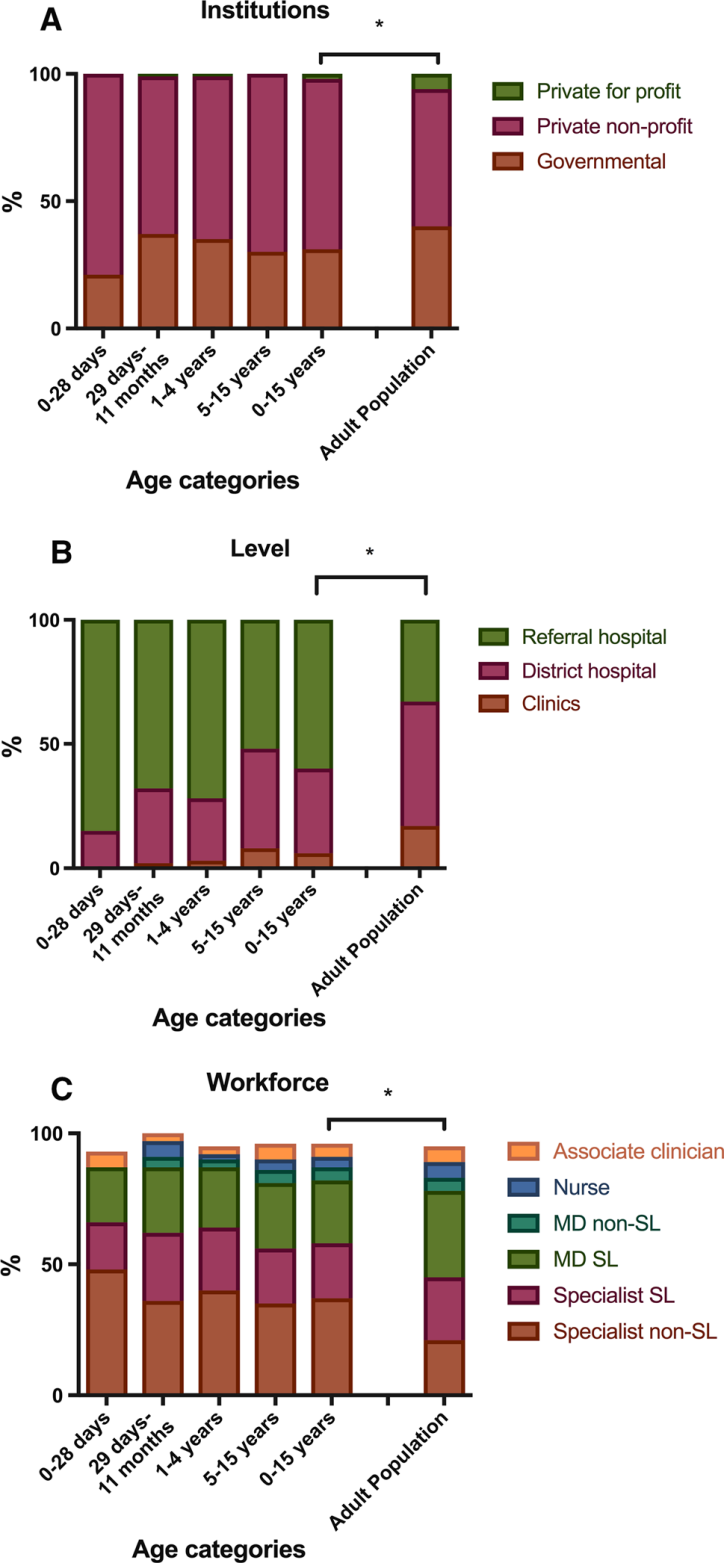
Age distribution of surgical procedures in Sierra Leone in 2012 by institutions (**a**), administration level (**b**) and workforce (**c**)

In 58 facilities with available surgical records out of 60, 40 performed pediatric surgery, of which 16 (40%) were governmental facilities, 19 (47%) private nonprofit and 5 (13%) private for-profit facilities. Almost one-third (31%) of the pediatric surgical procedures were performed in governmental facilities, and two-thirds (67%) were performed in private nonprofit facilities (Fig. 3a, Table 3). A total of 59% (1412) of the pediatric procedures were performed in referral, tertiary hospitals, and 39% (924) in district hospitals; the remaining 2% (45) were performed in clinics (Fig. 3b, Table 3). Significantly more children received surgical care at private nonprofit (*p* = 0.03) and referral hospitals (*p* < 0.0001) compared to the adult population.

**Table 3:** Surgical facilities and pediatric surgical procedures by owner, organizational level and workforce in Sierra Leone in 2012

	Facilities *n*/%	Procedures *n*/%
*Governmental*	14/35%	745/31.3%
Clinic	1/2.5%	2/0.08%
District hospital	9/22.5%	216/9.1%
Tertiary hospital	4/10%	527/22.1%
*Private nonprofit*	19/47.5%	1600/67.2%
Clinic	3/7.5%	7/0.4%
District hospital	14/35%	708/29.7%
Tertiary hospital	2/5%	885/37.1%
*Private for*-*profit*	6/15%	36/1.5%
Clinic	6/15%	36/1.5%
District hospital	0	0
Tertiary hospital	0	0
Total	40	2381

Over 67% of the pediatric surgeries were performed in private nonprofit institutions, and almost 50% in tertiary hospitals

Specialist surgeons performed 59% (1400) of the surgical procedures in children; of those, 42% (1002) were performed by a foreign surgical provider. The proportion of pediatric surgery performed by specialist and foreign surgical providers was higher in the youngest age-groups (Fig. 3c, Table 4). The proportion of non-Sierra Leonean workforce (*p* = 0.02) and specialist surgeons (*p* = 0.04) performing surgeries was significantly higher in the pediatric population compared to the adult population.

**Table 4:** Pediatric surgical procedures in Sierra Leone in 2012 by age category and workforce

Workforce	0–28 days *n*/(%)	29 days–11 months *n*/(%)	1–4 years *n*/(%)	5–15 years *n*/(%)	Total 0–15 years *n*/(%)
Specialist SL	6 (18.18%)	46 (26.7%)	173 (24%)	292 (21.1%)	517 (21.7%)
Specialist non-SL	16 (48.5%)	56 (36.6%)	294 (40.8%)	516 (35.5%)	883 (37.1%)
MD SL	7 (21.2%)	44 (25.6%)	167 (23.2%)	367 (25.2%)	585 (24.6%)
MD non-SL		8 (4.6%)	25 (3.5%)	86 (5.9%)	119 (5%)
Nurse		11 (6.4%)	18 (2.5%)	68 (4.7%)	97 (4%)
Associate clinician	2 (6.1%)	6 (3.5%)	25 (3.5%)	98 (6.7%)	131 (5.5%)
Unknown	1 (3%)	1 (0.6%)	19 (2.6%)	28 (1.9%)	49 (2.1%)
Total	33 (1.4%)	172 (7.2%)	721 (30.3%)	1455 (60.1%)	2381

**Fig. 4:**
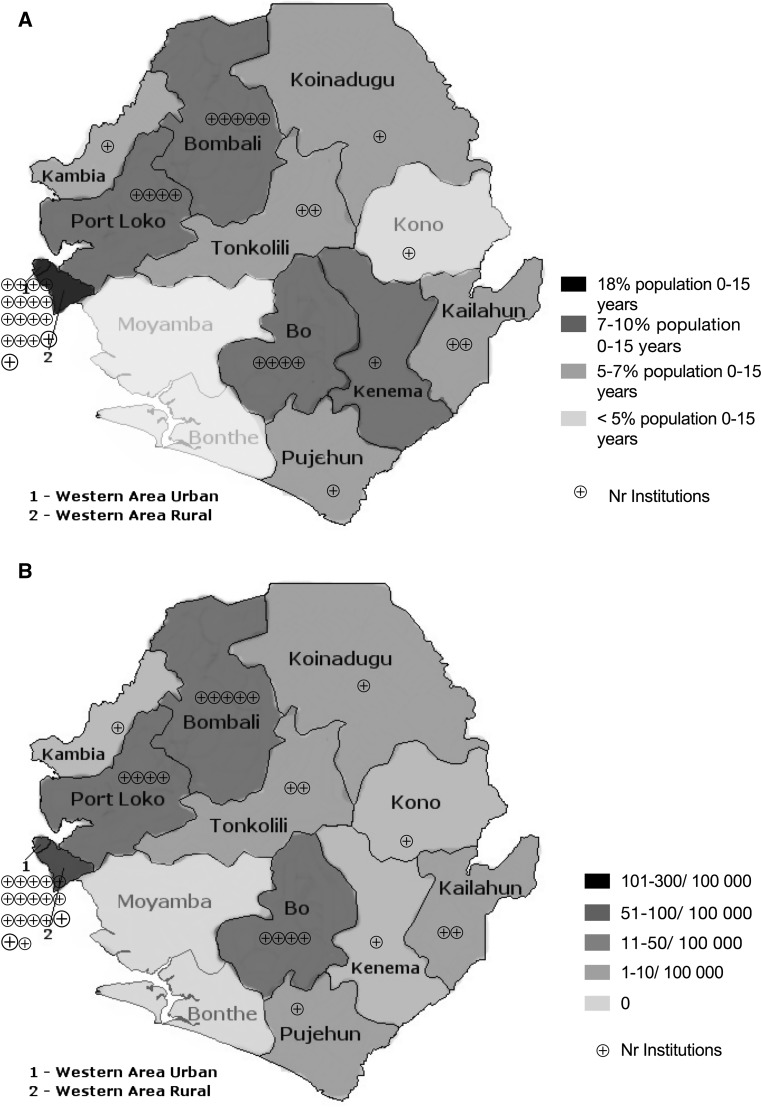
Map with the proportion of pediatric population (0–15 years) living in each district (**a**) and with the location of the facilities providing surgery in Sierra Leone 2012 and rate of pediatric surgery per 100,000 by district (**b**)

The rates of pediatric surgical procedures varied significantly between districts, ranging from 1 up to 290 procedures/100,000 inhabitants age 0–15 years (median 23/100,000 inhabitants aged 0–15 years). Two districts (Bonthe and Moyamba), accounting for 2.5 and 4%, respectively of the pediatric population, did not perform any pediatric surgery in 2012. More than half of the pediatric procedures were performed in the Western Area, a district where only 18% of the population under 16 years lives (Fig. 4a, b, Table 5). The number of facilities performing pediatric surgery varied also among the districts, ranging from 0 to 17 facilities (Fig. 4a, b, Table 5). In the Western Area, there were a total of 17 facilities performing surgery for children (44% of all facilities), accounting for 65% of the facilities performing pediatric surgery in the country. Most of the surgical procedures performed in the Western Area were performed in two tertiary hospitals, one governmental and one nonprofit hospital, accounting for 18 and 36%, respectively, of all surgical procedures performed in children in the country. In the remaining facilities, the volumes of pediatric surgical procedures ranged between 0.1 and 9% (Fig. 4a, Table 5).

**Table 5:** Rates of pediatric surgical procedures by district, age and gender (surgeries/100,000) in Sierra Leone in 2012, in relation to the pediatric population living in each district

Districts	Population							Rates of surgical procedures								
	0–4 years	Male 0–4y	Female 0–4y	5–15 years	Male 5–15y	Female 5–15y	% 0–15 years	Surgeries/100,000 0–4 years	Male 0–4y	Female 0–4y	Surgeries/100,000 5–15 years	Male 5–15y	Female 5–15y	Surgeries/100,000 0–15 years	Male 0–15y	Female 0–15y
Bo	97,611	49,100	48,511	184,351	88,486	95,864	9.4	91.2	132.4	49.5	68.9	70.1	66.8	76.6	92.3	60.9
Bombali	76,831	38,757	38,074	151,495	76,130	75,365	7.6	74.2	116.1	31.5	93.7	102.5	80.9	87.2	107.1	64.4
Kailahun	78,214	38,534	39,680	132,126	65,966	66,160	7.0	5.1	7.8	2.5	20.4	27.3	13.6	14.7	20.1	9.4
Kambia	62,064	31,284	30,780	104,955	53,616	51,339	5.6	1.6	3.2	0	1.9	1.9	1.9	1.8	2.4	1.2
Kenema	102,684	50,578	52,106	177,558	87,370	90,188	9.3	5.8	7.9	3.8	5.6	1.1	10	5.7	3.6	7.7
Koinadugu	51,578	25,283	26,295	104,931	51,412	53,519	5.2	7.8	15.8	0	14.3	17.5	11.2	12.1	16.9	7.5
Kono	51,049	25,665	25,384	84,260	41,428	42,832	4.5	1.9	0	0	1.2	2.4	0	1.5	1.5	0
Port Loko	96,546	49,431	47,115	164,559	84,370	80,188	8.7	23.8	26.3	10.6	85.1	65.2	82.3	62.4	50.8	55.8
Pujehun	60,528	29,791	30,737	90,462	46,444	44,018	5.1	33.1	50.4	0	16.6	19.4	9.1	23.2	31.5	5.4
Tonkolili	75,908	38,930	36,978	129,843	65,837	64,006	6.8	50.1	66.8	32.5	86.3	95.7	76.6	72.9	84.9	60.4
Western Area	164,199	85,227	78,972	368,550	177,022	191,528	17.7	416.6	520.9	303.9	234.4	306.7	159.8	290.6	376.4	201.8
Median	76,831	38,757	38,074	132,126	65,966	66,160	7	23.8	26.3	3.8	20.4	27.3	13.6	23.2	31.5	9.4
Mean	83,383	42,053	41,330	153,917	76,189	77,728	7.9	64.6	86.1	39.5	57.1	64.5	46.5	59	71.6	43.1

Large variation of rates of pediatric surgeries among districts, from 1 up to 290 surgeries/100,000, with a mean of 59 and a median 23/100,000

The expected new cases of inguinal hernia during 2012 (incidence 10–20/1000 births) based on the number of births (228,000) [41], were estimated to be between 2280 and 4558, average 3419 cases. Only 434 pediatric inguinal hernia repairs were performed during 2012 (Table 6).

**Table 6:** Incidence, cumulative incidence, met and unmet needs of pediatric inguinal hernia repair in Sierra Leone 2012

Sierra Leone	Male 0–15 years	Female 0–15 years	Both 0–15 years
Population	1,404,048	1,404,270	2,808,318
*Nr births 2012*			228,000
*Needs Inguinal Hernia repair*			
Annual Incidence (nr cases)	3078	341	3,419
Incidence/100 000 population	219	24	122
Prevalence/100 000 population	75,825	8425	84,250
Cumulative childhood incidence (nr cases)	20,315	239	20,554
Cumulative childhood incidence/100 000 population	1447	17	732
*Met needs Inguinal Hernia repair*			
Volume	379	55	434
Rate (per 100 000 population)	27	4	15
Met needs (%)	12	16	12
*Unmet needs Inguinal Hernia Repair*			
Volume (nr cases)	20,290	235	20,539
Rate (per 100 000 population)	192	21	106
Unmet needs (%)	88	84	88
Cumulative unmet needs (%)	98	87	94

The countrywide unmet pediatric surgical needs for inguinal hernia repair were calculated to be in the range of 88% in the pediatric population.

## Discussion

In 2010, the Sierra Leonean government launched a plan for free health care for children under 5 years of age, which had a tremendous impact on increasing access to surgical care by 500% in a tertiary hospital in the capital area [21]. This healthcare initiative has most likely affected the results of our study, at least in the capital area, but less so in rural areas.

As hypothesized, there is insufficient provision of safe pediatric surgical care in Sierra Leone, with rates of 84 surgical procedures/100,000 population 0–15 years. The most common surgical procedure among children in Sierra Leone is hernia repair, but at rates that cannot meet the increasing backlog of accumulated unrepaired hernias, despite the fact that pediatric inguinal hernia has been identified as one of the cost-effective 44 essential surgical procedures [43]. Moreover, more than half of the boys had their hernia repair done after 5 years of age. This delay in surgical repair will probably have led to a higher number of avertable incarcerations and complications [44]. The limited access to safe pediatric anesthesia, together with the higher risk of anesthesia at younger ages is likely an important contributor to this observed delay. There is an unequal distribution in the rates of pediatric surgeries and number of facilities among districts, with more than half of the procedures performed at only two referral hospitals in the Western Area. Compared to adults [36], children are more commonly operated in referral hospitals and by specialist surgeons, likely because surgery at younger ages carries greater risks and is technically more challenging. One-third of all the pediatric procedures were performed in a single private nonprofit hospital and 40% by non-Sierra Leonean provider. This highlights the lack of domestic capacity for pediatric surgery. Our estimate indicates that only one out of ten children in Sierra Leone gets the essential surgery he or she needs, consistent with previous reports [2]. Our results are compounded by the increasing backlog of untreated surgical disease that accumulates each year. However, the true unmet need for pediatric surgery, including more complex surgery, is probably considerably higher.

It may be reasonable to centralize pediatric surgery to hospitals with competencies to provide safe pediatric anesthesia. On the other hand, a centralized service will exclude rural population and acute conditions, since the main barrier to access surgical care in Sierra Leone is financial [45], where indirect cost such as travel is a major contributor [2]. Long distance to facilities providing surgical services has been reported to affect negatively the access to pediatric surgical treatments [46]. Delayed health-seeking behavior at a patient level [47, 48], limitations in infrastructure as a second level of delay [48] and workforce shortages [48, 49], all could contribute to the limited number of surgical procedures performed among Sierra Leonean children. Moreover, some of the direct and indirect effects of the Ebola outbreak in 2014 on health workers have resulted in an aggravated shortage in the workforce and fatalities among students enrolled, a profound decline in hospital admissions and surgical procedures performed since have all contributed [50].

Despite the fact that surgical procedures are cost-effective [51], only 10% of adults [36] and children get the surgery they need. Procedures performed at first-level hospitals have been found to be the most cost-effective [51], and surgical repair of pediatric inguinal hernia has been shown to be highly cost-effective [52]. The Lancet Commission on Global Surgery recently stratified common surgical procedures that must be done, should be done and can be done in first-level hospitals [53]. Some of these procedures for the pediatric population include laparotomies, fracture treatment, cesarean section, hernia and cleft lip repair.

There are several limitations with this study. Limitations related to data collection and completeness of number of surgeries in the country have been discussed in a previous work [36], and include the retrospective nature of the study and data collection from different registries, as well as facility categorization, possible inconsistencies or variations in recording routines, the assumption when calculating district or facilities rates that patients get the surgery done in the district where they live. Another limitation of the study is the standardized way the data were collected, in order to make possible comparisons and conclusions over a lifespan, but with a large proportion of unspecified “other” procedures. Furthermore, we cannot accurately know the number of hernias that were incarcerated or the number of complications. Only 19 out of 434 surgical hernia repairs were registered as emergency cases, which may indicate incarceration. Incarcerated inguinal hernias with devastating consequences such as bowel necrosis could have also been registered as general surgery other or laparotomies.

Our estimation of unmet pediatric surgical needs also has limitations. Currently, there is no well-established method to define the need for pediatric surgery. Some household surveys have defined surgical needs based on patient’s own judgment, with the potential of overestimations [34]. The Lancet Commission for Global Surgery estimated the global need of surgery based on the known prevalence of conditions that are primarily treated with surgery [4]. Others have used surgical tracers such as groin hernia and ratio of cesarean section [27]. Even though inguinal hernia seems to be a reliable tracer to estimate the surgical needs in the pediatric population, this calculation also has its limitations, since it assumes that the incidence of inguinal hernia is similar among populations. We calculated the expected incidence of new cases of inguinal hernia in 2012 based on the number of births in 2012 and tried to estimate the cumulative incidence, but our study design does not allow calculation of the real prevalence of inguinal hernia in the pediatric population. Our analysis is limited to the provision of surgical procedures in 2012 and does not take into account possible targeted surgical interventions in the country in the previous years. Even if the estimation of the unmet pediatric surgical needs based on the incidence of inguinal hernia has its limitations, currently there are few other alternatives.

Despite potential underestimation, our results suggest that only 10% of the children in Sierra Leone get the surgery they need. This lack of pediatric surgical care needs to be urgently addressed. The coverage of 44 defined essential surgical procedures [51] could avert deaths attributable to surgically treatable conditions in the country, making increased access to safe surgery an urgent need. Globally, surgically treatable conditions account for 10% of all deaths annually (5 millions) [9, 54], but 1.5 million could be prevented [43, 51] with essential surgical procedures [51]. Surgical care could be provided in an increased number of facilities around the country by training and delegating certain responsibilities to less specialized workers, such as the provision of general anesthesia to anesthesia nurses (task sharing), or the shifting of tasks from surgeons to non-specialists or from doctors to non-doctors (task shifting) [55, 56]. A task-shifting program started in Sierra Leone in 2011, where community health officers with 3 years of basic medical education and 2 years of clinical experience received 3 years of essential surgical training, to manage common surgical conditions. Specific training in pediatric inguinal hernia and injuries, which are identified as a part of the cost-effective 44 essential surgical procedures, [43] should be implemented in the existing task-shifting program in the country.

Estimating the unmet surgical need can guide policy makers in resource-scarce settings such as Sierra Leone. Surgical conditions likely cause a significant burden of disease in the pediatric population, and pediatric surgery should not remain a neglected public health service in the country.

## Conclusions

- Ten percent of all surgical procedures performed in Sierra Leone in 2012 were performed in children, corresponding to a total of 2381 surgical procedures.
- The rate of pediatric surgical procedures was 84 per 100,000.
- In 2012, inguinal hernia was the most common surgical procedure performed in children in Sierra Leone.
- Most of the pediatric surgical procedures were performed in the capital area, in private non-for-profit tertiary hospitals and by consultant surgeons and non-Sierra Leonean workforce.
- There seems to be a considerable unmet need (almost 90%) for hernia repair as a proxy indicator for the needs of pediatric surgical care in Sierra Leone.

## Appendix

**Table:** List of 44 essential surgical procedures. Adopted from DCP 3, volume 1: *essential surgery* ^1^

Type of procedure	Platform for delivery		
	Primary health center	First-level hospital	Tertiary hospital
Dental procedures	1. Extraction 2. Drainage of dental abscess 3. Treatment for caries		
Obstetric, gynecologic and family planning	4. *Normal delivery*	1. *Cesarean section* 2. *Vacuum extraction/forceps delivery* 3. *Ectopic pregnancy* 4. *Dilation and curettage* 5. Tubal ligation 6. Vasectomy 7. *Hysterectomy for uterine rupture or intractable postpartum hemorrhage* 8. Visual inspection with acetic acid and cryotherapy for precancerous cervical lesions	1. Repair obstetric fistula
General surgery	5. *Drainage superficial abscess* 6. Male circumcision	9. *Repair of perforations: perforated peptic ulcer, typhoid ileal perforation,* etc. 10. *Appendectomy* 11*. Bowel obstruction* 12. *Colostomy* 13. Gallbladder disease *(including emergency surgery for acute cholecystitis)* 14. Hernia *(including incarceration)* 15. Hydrocelectomy 16. *Relief of urinary obstruction: catheterization or suprapubic cystostomy (tube into bladder through skin)*	
Trauma	7. *Resuscitation with basic life support measures* 8. *Suturing laceration* 9. *Management of non*-*displaced fracture*	17*. Resuscitation with advanced life support measures, including surgical airway* 18. *Tube thoracostomy (chest* drain) 19. *Trauma laparotomy* 20. *Fracture reduction* 21*. Irrigation and debridement of open fractures* 22*. Placement of external fixator, use of traction* 23*. Escharotomy/fasciotomy (cutting of constricting tissue to relieve pressure from swelling)* 24*. Trauma*-*related amputations* 25. Skin grafting 26. *Burr hole*	
Congenital			2. Cleft lip and palate repair 3. Club foot repair 4. Shunt for hydrocephalus 5. Repair of anorectal malformations and Hirschsprung’s disease
Ophthalmology			6. Cataract extraction and insertion of intraocular lens 7. Eyelid surgery for trachoma
Orthopedic		27*. Drainage of septic arthritis* 28. *Debridement of osteomyelitis*	

^1^ Alkire B VJ, Meara J. Chapter 21. Benefit-Cost Analysis for Selected Surgical Interventions in Low and Middle Income Countries. In: Debas HT, Donkor P, Gawande A, Jamison DT, Kruk ME, Mock CN, eds. Disease Control Priorities: Third Edition Volume 1 Essential Surgery: World Bank; 2015
